# CT-Based Pelvic T_1_-Weighted MR Image Synthesis Using UNet, UNet++ and Cycle-Consistent Generative Adversarial Network (Cycle-GAN)

**DOI:** 10.3389/fonc.2021.665807

**Published:** 2021-07-30

**Authors:** Reza Kalantar, Christina Messiou, Jessica M. Winfield, Alexandra Renn, Arash Latifoltojar, Kate Downey, Aslam Sohaib, Susan Lalondrelle, Dow-Mu Koh, Matthew D. Blackledge

**Affiliations:** ^1^Division of Radiotherapy and Imaging, The Institute of Cancer Research, London, United Kingdom; ^2^Department of Radiology, The Royal Marsden Hospital, London, United Kingdom; ^3^Gynaecological Unit, The Royal Marsden Hospital, London, United Kingdom

**Keywords:** convolutional neural network (CNN), generative adversarial network (GAN), medical image synthesis, radiotherapy planning, magnetic resonance imaging (MRI), computed tomography (CT)

## Abstract

**Background:**

Computed tomography (CT) and magnetic resonance imaging (MRI) are the mainstay imaging modalities in radiotherapy planning. In MR-Linac treatment, manual annotation of organs-at-risk (OARs) and clinical volumes requires a significant clinician interaction and is a major challenge. Currently, there is a lack of available pre-annotated MRI data for training supervised segmentation algorithms. This study aimed to develop a deep learning (DL)-based framework to synthesize pelvic T_1_-weighted MRI from a pre-existing repository of clinical planning CTs.

**Methods:**

MRI synthesis was performed using UNet++ and cycle-consistent generative adversarial network (Cycle-GAN), and the predictions were compared qualitatively and quantitatively against a baseline UNet model using pixel-wise and perceptual loss functions. Additionally, the Cycle-GAN predictions were evaluated through qualitative expert testing (4 radiologists), and a pelvic bone segmentation routine based on a UNet architecture was trained on synthetic MRI using CT-propagated contours and subsequently tested on real pelvic T_1_ weighted MRI scans.

**Results:**

In our experiments, Cycle-GAN generated sharp images for all pelvic slices whilst UNet and UNet++ predictions suffered from poorer spatial resolution within deformable soft-tissues (e.g. bladder, bowel). Qualitative radiologist assessment showed inter-expert variabilities in the test scores; each of the four radiologists correctly identified images as acquired/synthetic with 67%, 100%, 86% and 94% accuracy. Unsupervised segmentation of pelvic bone on T1-weighted images was successful in a number of test cases

**Conclusion:**

Pelvic MRI synthesis is a challenging task due to the absence of soft-tissue contrast on CT. Our study showed the potential of deep learning models for synthesizing realistic MR images from CT, and transferring cross-domain knowledge which may help to expand training datasets for 21 development of MR-only segmentation models.

## Introduction

Computed tomography (CT) is conventionally used for the delineation of the gross tumor volume (GTV) and subsequent clinical/planning target volumes (CTV/PTV), along with organs-at-risk (OARs) in radiotherapy (RT) treatment planning. Resultant contours allow optimization of treatment plans by delivering the required dose to PTVs whilst minimizing radiation exposure of the OARs by ensuring that spatial dose constraints are not exceeded. Magnetic resonance imaging (MRI) offers excellent soft-tissue contrast and is generally used in conjunction with CT to improve visualization of the GTV and surrounding OARs during treatment planning. However, manual definition of these regions is repetitive, cumbersome and may be subject to inter- and/or intra-operator variabilities (1). The recent development of the combined MR-Linac system (2) provides the potential for accurate treatment adaption through online MR-imaging acquired prior to each RT fraction. However, re-definition of contours for each MR-Linac treatment fraction requires approximately 10 minutes of downtime whilst the patient remains on the scanner bed, placing additional capacity pressures on clinicians wishing to adopt this technology.

Deep learning (DL) is a sub-category of artificial intelligence (AI), inspired by the human cognition system. In contrast to traditional machine learning approaches that use hand-engineered image-processing routines, DL is able to learn complex information from large datasets. In recent years, DL-based approaches have shown great promise in medical imaging applications, including image synthesis (3, 4) and automatic segmentation (5–7). There is great promise for DL to drastically accelerate delineation of the GTV and OARs in MR-Linac studies, yet a major hurdle remains the lack of large existing pre-contoured MRI datasets for training supervised segmentation networks. One potential solution is transferring knowledge from pre-existing RT planning repositories on CT to MRI in order to facilitate domain adaptive segmentation (8). Previous studies have reported successful implementation of GANs in generating realistic CT images from MRI (3, 9–11) as well as MRI synthesis from CT in the brain (12). To date, few studies have investigated MRI synthesis in the pelvis. Dong et al. (13) proposed a synthetic MRI-assisted framework for improved multi-organ segmentation on CT. However, although the authors suggested that synthetic MR images improved segmentation results, the quality of synthesis was not investigated in depth. MR image synthesis from CT is a challenging task due to large soft-tissue signal intensity variations. In particular, MRI synthesis in the pelvis offers the considerable difficulty posed by geometrical differences in patient anatomy as well as unpredictable discrepancies in bladder and bowel contents.

In this study, we compare and contrast paired and unpaired generative techniques for synthesizing T_1_-weighted (T_1_W) MR images from pelvic CT scans as a basis for training algorithms for OARs and tumor delineation on acquired MRI datasets. We include in our analysis the use of state-of-the-art UNet (14) and UNet++ (15) architectures for paired training, testing two different loss functions [L_1_ and VGG-19 perceptual loss (16)], and compare our results with a Cycle-Consistent Generative Adversarial Network (Cycle-GAN) (17) for unpaired MR image synthesis. Subsequently, we evaluate our results through blinded assessment of synthetic and acquired images by expert radiologists, and demonstrate our approach for pelvic bone segmentation on acquired T_1_W MRI from a framework trained solely on synthetic _1_W MR images with CT-propagated contours.

## Materials and Methods

### Patient Population and Imaging Protocols

Our cohort consisted of 26 patients with lymphoma who underwent routine PET/CT scanning (Gemini, Philips, Cambridge, United States) and whole-body T_1_W MRI (1.5T, Avanto, Siemens Healthcare, Erlangen, Germany) before and after treatment (see **Table 1** for imaging protocols). From this cohort, image series with large axial slice angle mismatch between CT and MR images, and those from patients with metal implants were excluded, leaving 28 paired CT/MRI datasets from 17 patients. The studies involving human participants were reviewed and approved by the Committee for Clinical Research at the Royal Marsden Hospital. The patients/participants provided written informed consent to participate in this study.

**Table 1:** Imaging parameters for acquired CT and T_1_W MR images.

CT parameters		T_1_W MR parameters	
Peak Voltage Output (kVp)	120	Acquisition Sequence	2D Spoiled Gradient Echo
Acquisition Type	Helical	Echo Time (ms)	4.8
Slice Thickness (mm)	3-6.5	Repetition Time (ms)	386
Matrix Size	512 *×* 512	Phase Encoding Direction	Anterior-Posterior
Pixel Spacing (mm^2^)	0.74 *×* 0.74-1.17 *×* 1.17	Acquired Matrix Size (read)	256
Exposure (mAs)	26-80	Reconstructed Matrix Size (read)	512
		Reconstructed Pixel Size (mm^2^)	0.74 *×* 0.74-0.82 *×* 0.82
		Flip Angle	70°
		Slice Orientation	Axial
		Slice Thickness (mm)	5
		Acceleration	GRAPPA (R=2)
		Bandwidth	Pixel
		(Hz/pixel)	331

Some parameters are shown as the range of values (minimum-maximum) existing in the patient datasets.

### Model Architectures

**Figure 1:**
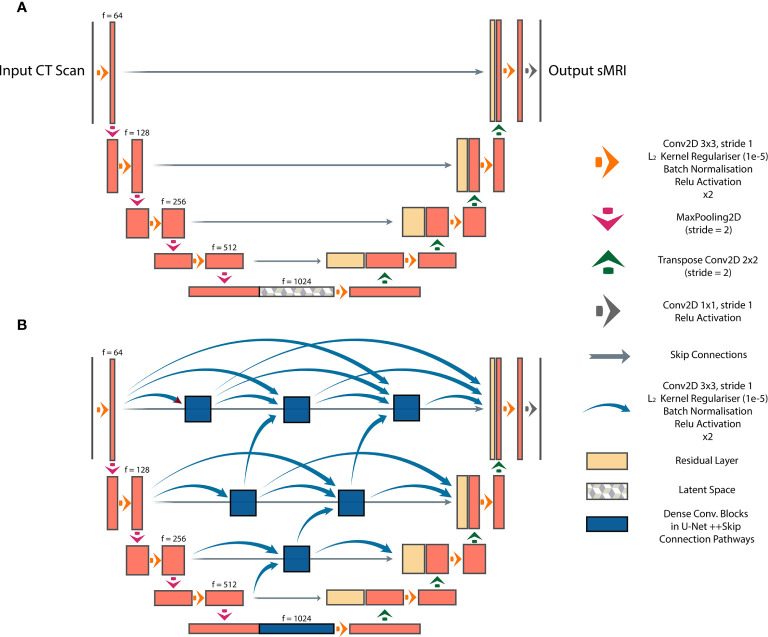
Paired image-to-image networks, **(A)** UNet with symmetrical skip connections between the encoder and decoder, **(B)** UNet++ with interconnected skip connection convolutional pathways.

We investigated three DL architectures for MR image synthesis: (i) UNet, (ii) UNet++, and (iii) Cycle-GAN. UNet is one of the most popular DL architectures for image-to-image translations, with initial applications in image segmentation (14). In essence, UNet is an auto-encoder with addition of skip connections between encoding and decoding sections to maintain spatial resolution. In this study, a baseline UNet model was designed consisting of 10 consecutive convolutional blocks (5 encoding and 5 decoding blocks), each using batch normalization and ReLU activation for CT-to-MR image generation (**Figure 1A**). Additionally, a UNet++ model with interconnected skip connection pathways, as described in (15), was developed with the same number of encoder-decoder sections and kernel filters as the baseline UNet (**Figure 1B**). UNet++ was reported to enhance performance (15), therefore we deployed this architecture to assess its capabilities for paired image synthesis.

**Figure 2:**
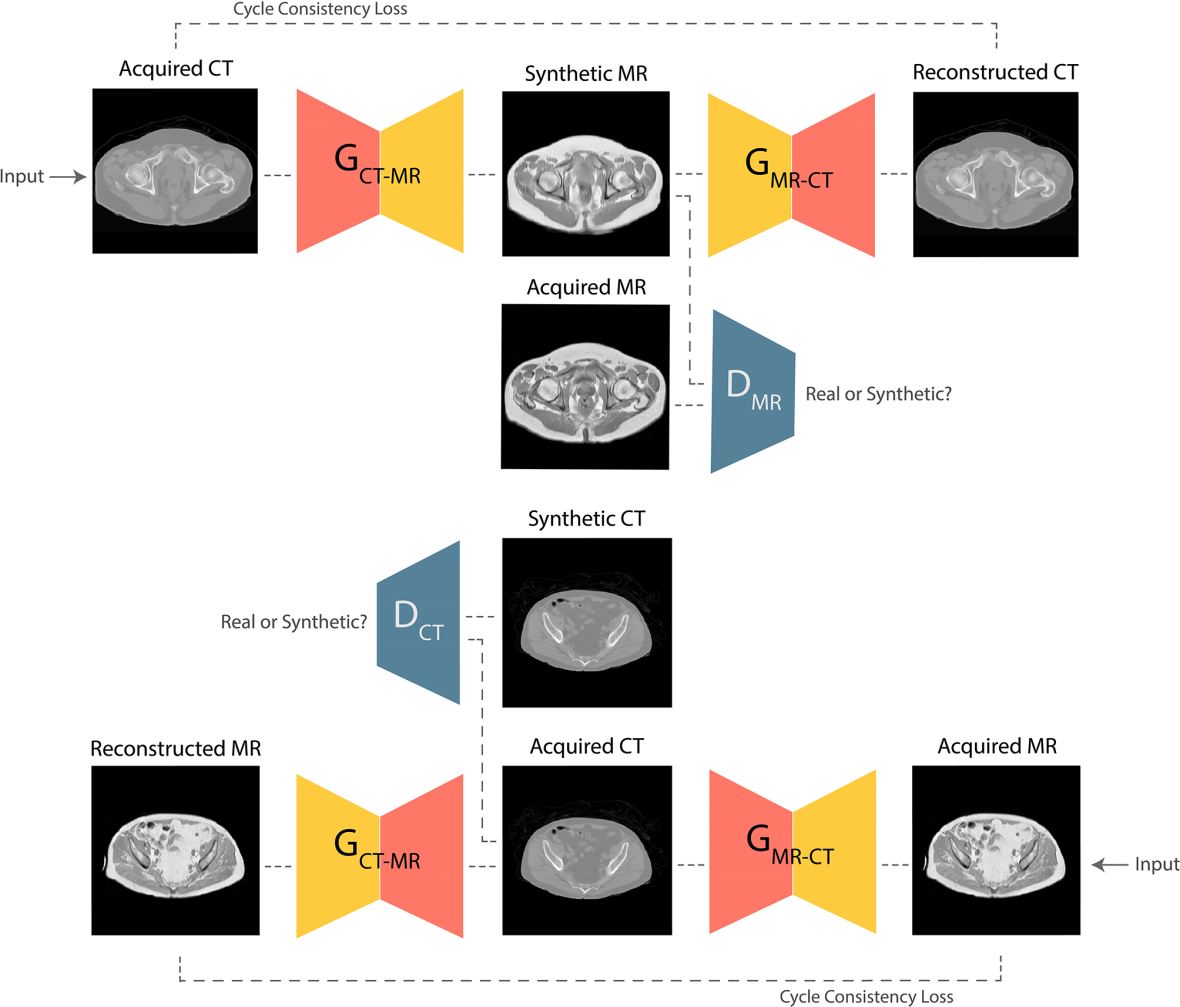
Schematic of the Cycle-GAN network. During training, images from CT domain are translated to MRI domain and reconstructed back to CT domain under the governance of adversarial and cycle consistency loss terms respectively. Co-training of CT-to-MRI and MRI-to-CT models leads to generation of photo-realistic predictions.

GANs are the state-of-the-art approaches for generating photo-realistic images based on the principles of game theory (18). In image synthesis applications, GANs typically consist of two CNNs, a generator and a discriminator. During training, the generator produces a target synthetic image from an input image with different modality; the discriminator then attempts to classify whether the synthetic image is genuine. Training is successful once the generator is able to synthesize images that the discriminator is unable to differentiate from real examples. Progressive co-training of the generator and discriminator leads to learning of the global conditional probability distribution from input to target domain. In this study, a Cycle-GAN model (17) was developed to facilitate unpaired CT-to-MR and MR-to-CT learning. The baseline UNet model was used as the network generator, and the discriminator composed of 5 blocks containing 2D convolutional layers followed by instance normalization and leaky ReLU activation. This technique offers the advantage that it does not require spatial alignment between training T_1_W MR and CT images. The high-level schematic of the Cycle-GAN network is shown in **Figure 2**.

**Figure 3:**
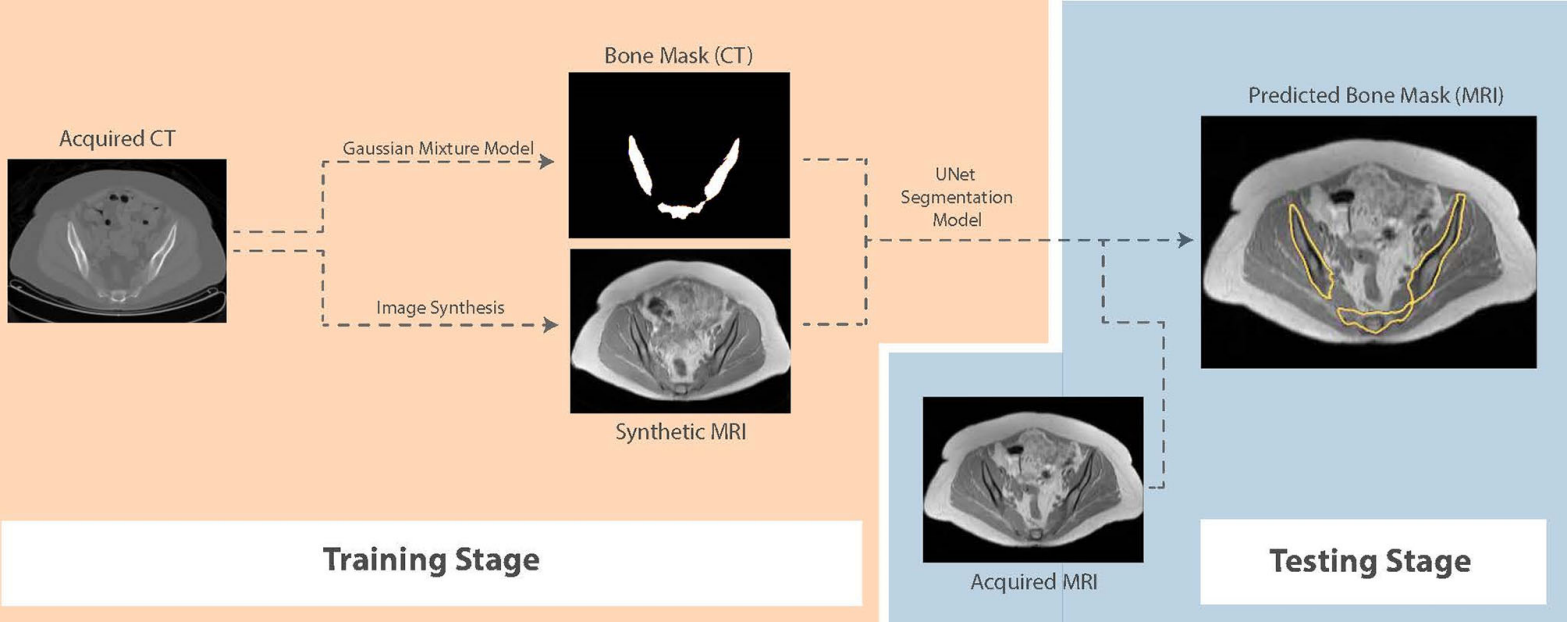
Schematic of the proposed fully-automatic combined synthesis and segmentation framework for knowledge transfer from CT scans to MR images. The intermediate synthesis stage enables segmentation training using CT-based contours and MR signal distributions.

For segmentation, we propose a framework that first generates synthetic T1W MR images from CT, propagates ground-truth CT contours and outputs segmentation contours on acquired T1W MR images. To examine the capability of our fully-automated DL framework for knowledge transfer from CT to MRI, we generate ground-truth contours of the bones using a Gaussian mixture model proposed by Blackledge etal. (19) and transfer them to synthetic MR images as a basis for our segmentation training. A similar UNet model to the architecture presented in **Figure 1**, with 5 convolutional blocks (convolution-batchnorm-dropout(p=0.5)-ReLU) in the encoding and decoding sections was developed to perform binary bone segmentation from synthetic MR images. The schematic of our proposed synthesis/segmentation framework is illustrated in **Figure 3**.

### Image Preprocessing

In preparation for paired training, the corresponding CT and T_1_W MR slices from the anatomical pelvic station in each patient were resampled using a 2D affine transformation followed by non-rigid registration using multi-resolution B-Spline free-form deformation (loss = Mattes mutual information, histogram bins = 50, gradient descent line search optimizer parameters: learning rate = 5.0, number of iterations = 50, convergence window size = 10) (20). The resulting co-registered images were visually qualified based on the alignment of rigid pelvic landmarks. In CT images, signal intensities outside of the range -1000 and 1000 HU were truncated to limit the dynamic range. The T_1_W MR images were corrected using N4 bias-field correction to reduce inter-patient intensity variations and inhomogeneities (21) and signal intensities above 1500 (corresponding to infrequent high intensity fatty regions) were truncated. Subsequently, the training images were normalized to intensity ranges (0,1) and (-1,1) prior to paired (UNet, UNet++) and unpaired (Cycle-GAN) training respectively.

### Objective Functions

Common loss functions in image synthesis are mean absolute error (MAE or L_1_) and mean squared error (MSE or L_2_) between the target domain and the synthetic output. However, such loss functions ignore complex image features such as texture and shape. Therefore, for UNet/UNet++ models, we compared L_1_ loss in the image space with L_1_ loss calculated based on the features extracted from a previously-trained object classification network, deriving the “perceptual loss”. For this purpose, the VGG-19 classification network was used (16), which is composed of 5 convolutional layers and 19 layers overall, and used features extracted from the “block conv2d” layer. For Cycle-GAN training, the difference between L_1_ and the structural similarity index (SSIM) (defined as L_1_ – SSIM) was used as the loss to govern the cycle consistency, whilst L_1_ and L_2_ losses were used for the generator and the discriminator respectively. For segmentation training, the Dice loss (1, 2) was used to perform binary division of bone on MR images.

(1) $$DSC=\frac{2|A\cap B|}{\left|A|+|B\right|}$$

(2) Dice loss=1−DSC

where A and B denote the generated and ground-truth contours.

### Model Training and Evaluation

The dataset was split to 981, 150 and 116 images from 11, 3 and 3 patients for training, validation and testing respectively. All models were trained for 150 epochs using the Adam optimizer (learning rate = 1e-4; UNet and UNet++ models: batch size = 5, Cycle-GAN: batch size=1) on a NVIDIA RTX6000 GPU (Santa Clara, California, United States) (**Table 2**). During paired UNet/UNet++ training, the peak signal-to-noise ratio (PSNR), SSIM, L_1_ and L_2_ quantitative metrics, as described in (22), were recorded at each epoch for the validation images. The trained weights with the lowest validation loss were used to generate synthetic T_1_W MR images from the test CT images. Optimal weights from the Cycle-GAN model were selected based on visual examination of the network predictions of the validation data following each epoch. Subsequently, synthetic images from all models were evaluated against the ground-truth acquired MR images quantitatively using the above-mentioned imaging metrics. An additional qualitative test was designed to obtain unbiased clinical examination of predictions from the Cycle-GAN model. This test consisted of two sections: (i) to blindly classify randomly-selected test images as synthetic or acquired, and outline reasoning for answers (18 synthetic and 18 acquired test MR slices), and (ii) to describe key differences between synthetic and acquired test T_1_W MR images when the input CT and ground truth acquired MR images were also provided (10 sets of images from 3 test patients). This test was completed by 4 radiologists (two with *<*5 years and two with *>*5 years of experience). The segmentation network was trained on Cycle-GAN generated synthetic MR images (training: 14, validation: 3 patients) for 600 epochs using the Adam optimizer (learning rate = 1e-4) and batch size of 1. To avoid overfitting, random linear shear and rotation (range:0, *π*/60) were applied to images during training.

**Table 2:** Learnable parameters (in millions) of UNet, UNet++ and Cycle-GAN models.

	UNet (L_1_)	UNet (VGG)	UNet++ (L_1_)	Cycle-GAN
Trainable Parameters (M)	31	31	36	31(G), 11(D)

G and D denote the Cycle-GAN generator and discriminator respectively.

## Results

**Figure 4:**
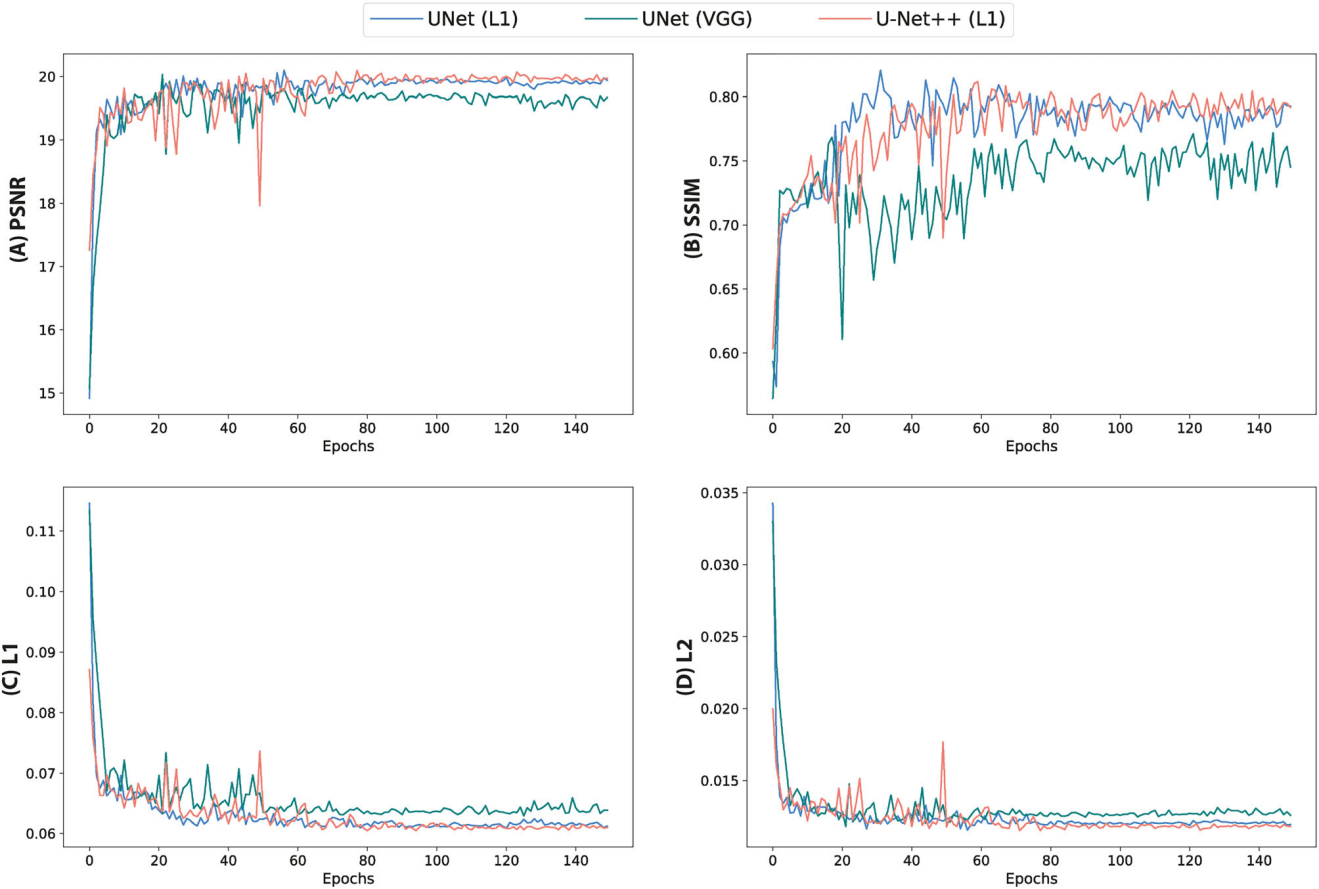
Quantitative metrics calculated from validation images during training of UNet and UNet++ models for 150 epochs. **(A)** PSNR, **(B)** SSIM, **(C)** L_1_ loss and **(D)** L_2_ loss.

**Figure 5:**
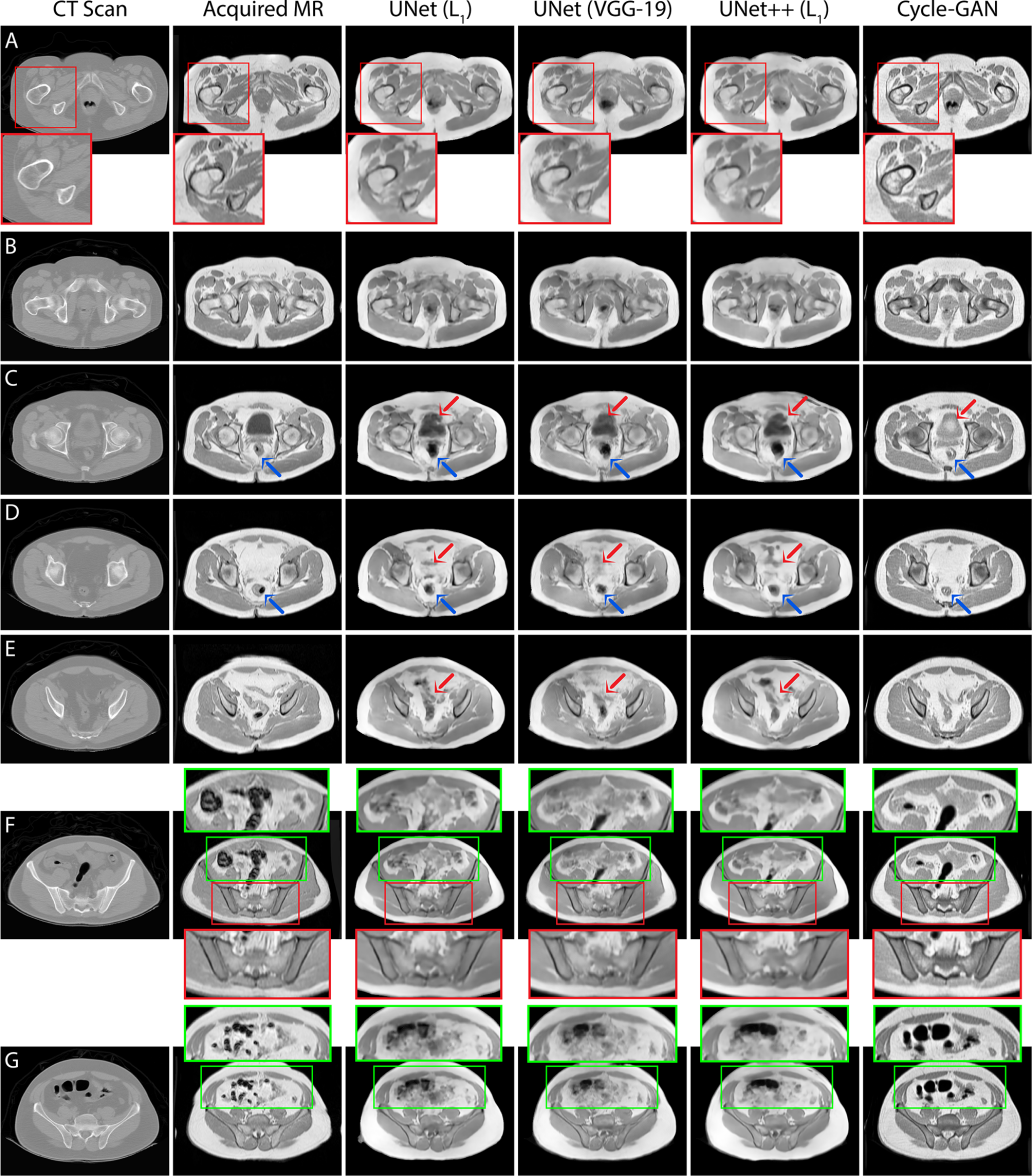
T_1_T1W MRI predictions generated from 3 independent test patients using UNet, UNet++ and Cycle-GAN models (panel **A**: patient 1, panels **B–E**, **G**: patient 2, panel **F**: patient 3). Red box: Predictions from pelvic slices with relatively fixed geometries including the bones demonstrate sharp boundaries between anatomical structures, with visually superior results for the Cycle-GAN architecture (panels **A**, **F**). Green box: The superior resolution of the Cycle-GAN architecture is further exemplified in slices with deformable structures such as the bowel loop (panels **F**, **G**). In highly deformable regions, minor contrast disparity in anatomical structures can be observed in the synthetic MRI; examples include prediction of bladder (red arrows in panel **C**), lower gastrointestinal region (red arrows in panels **D**, **E**) and rectum (blue arrows in panels **C**, **D**).

Quantitative assessment of synthetic T_1_W MR images from the validation dataset during paired algorithm training suggested that the UNet and UNet++ models with L_1_ loss displayed higher PSNR and SSIM, and lower L_1_ and L_2_ values compared with the generated images from the UNet model with the VGG-19 perceptual loss (**Figure 4**). Quantitative analysis of synthetic images from the test patients revealed a similar trend for UNet and UNet++ model predictions and showed that the Cycle-GAN quantitative values were the lowest in all metrics but the SSIM where it was only higher than UNet (VGG) predictions (**Table 3**). Moreover, qualitative evaluation of predictions from all models revealed a noticeable difference in sharpness (spatial resolution) between the images generated from paired (UNet and UNet++) and unpaired (Cycle-GAN) training. It was observed that despite UNet and UNet++ models generating relatively realistic predictions for pelvic slices consisting of fixed and bony structures (e.g. femoral heads, hip bone, muscles), they yielded blurry and unrealistic patches for deformable and variable pelvic structures such as bowel, bladder and rectum. In contrast, the Cycle-GAN model generated sharp images for all pelvic slices, yet a disparity in contrast was observed for soft-tissues with large variabilities in training patient MRI slices (e.g. bowel content, gas in rectum and bowel, bladder filling) (**Figure 5**).

**Table 3:** Quantitative analysis of predictions from the trained models on test patients.

	UNet (L_1_)	UNet (VGG)	UNet++ (L_1_)	Cycle-GAN PSNR
**PSNR**	**20**.**169***±***0**.**196**	19.668 *±* 0.189	20.080 *±* 0.193	18.279 *±* 0.156
**SSIM**	**0**.**809***±***0**.**003**	0.728 *±* 0.003	0.804 *±* 0.003	0.783 *±* 0.003
**MAE**	**0**.**043***±***0**.**001**	0.047 *±* 0.001	0.044 *±* 0.001	0.057 *±* 0.001
**MSE**	**0**.**011***±***0**.**001**	**0**.**011***±***0**.**001**	0.013 *±* 0.001	0.016 *±* 0.001

The calculated metrics are presented as mean and standard deviation. The pixel intensities outside the body were excluded when deriving these measurements. The best quantitative metrics are shown in bold.

**Figure 6:**
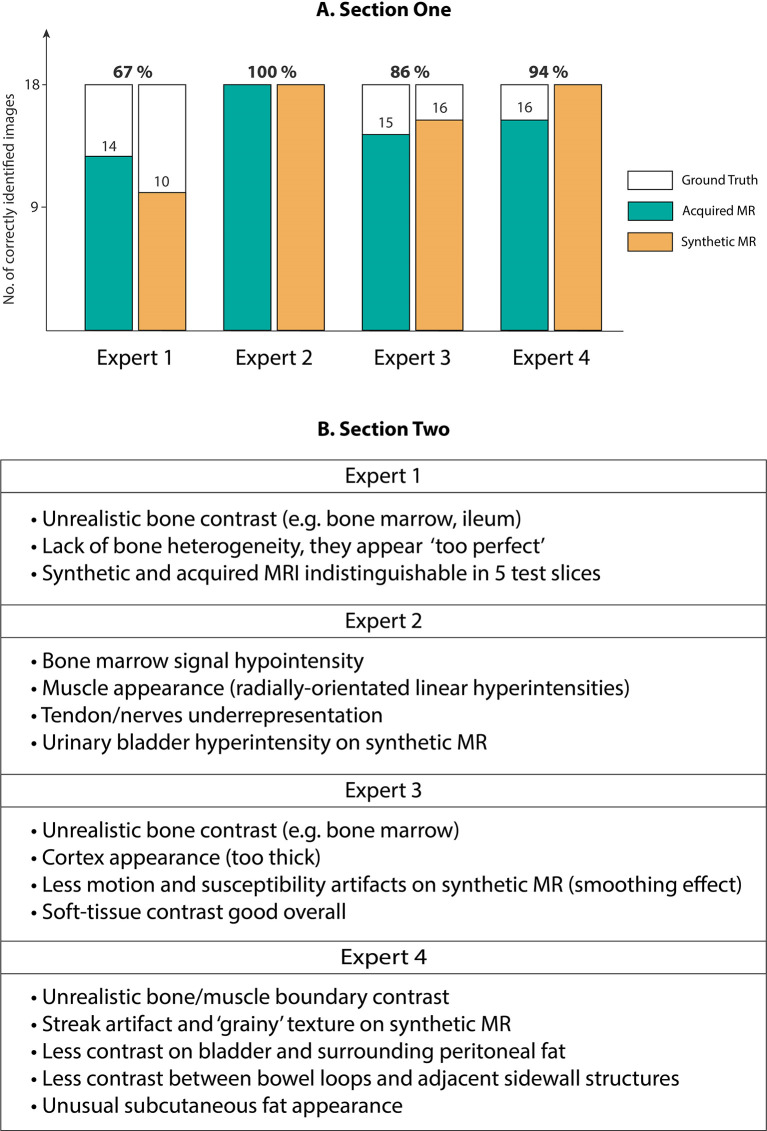
**(A)** Section One: Expert scores for identifying evenly-distributed test patient MRI slices as synthetic or acquired, **(B)** Section Two: Expert comments on Cycle-GAN synthetic MRI when presented along with the ground truth CT and acquired T_1_W MRI (Experts 1 and 2 with <5 years of experience, and experts 3 and 4 with >5 years of experience).

Our expert radiologist qualitative testing on Cycle-GAN predicted images suggested that there were inter-expert variabilities in scores from section one of the test, highlighting the differences in subjective decisions amongst the experts in a number of test images. Experts 1 and 2 (*<*5 years of experience) scored 67% and 100% whilst experts 3 and 4 (*>*5 years of experience) correctly identified 86% and 94% of total 36 test images. Hence, no particular correlation was observed between the percentage scores and the participants’ years of experience (**Figure 6A**). Radiologist comments on the synthetic images (following unblinding) are presented in **Figure 6B**.

**Figure 7:**
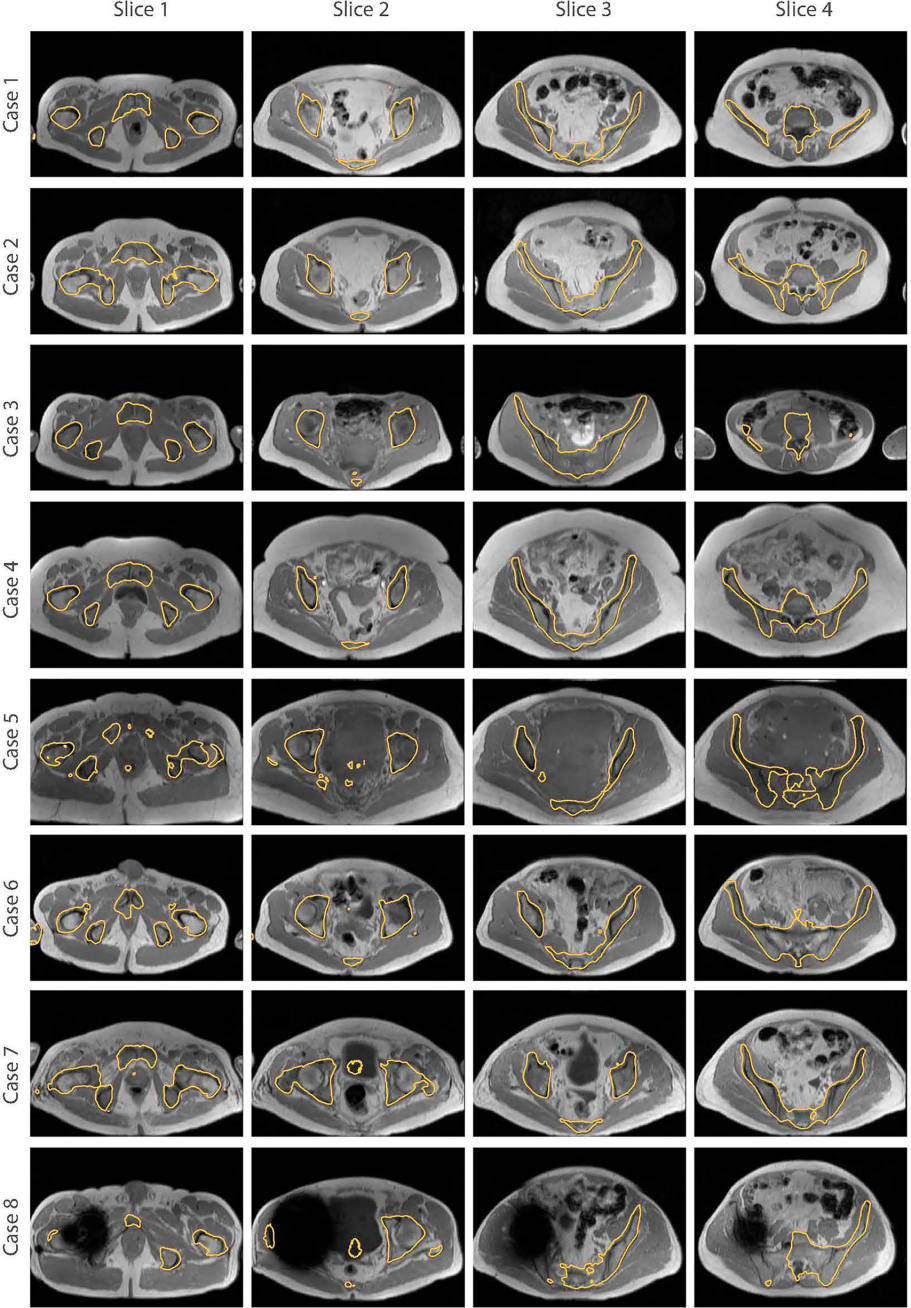
Bone segmentation results from acquired T_1_W MRI scans of 8 test patients using the proposed fully-automated framework. The combined synthesis/segmentation network allows transfer of organ- specific encoded spatial information from CT to MRI without the need to manually define ROIs. Cases 5 to 8 were patients not included in the synthesis stage of network training. Case 8 shows bone segmentation results from a patient with metal hip.

The bone segmentation results using our fully-automated approach showed that our proposed framework successfully performed unsupervised segmentation of the bone from acquired T_1_W MR images, without the requirement of any manually annotated regions of interest (ROIs). The outcome from various pelvic slices across 8 patients from our in-house cohort are presented in **Figure 7**. The segmentation results from cases 5 to 8 were from patients not used in the synthesis and segmentation components of our framework. Test case 8 demonstrates the predicted bone contours from a patient with metal hip implant.

## Discussion and Conclusion

One major limitation in adaptive RT on the MR-Linac system is the need for manual annotation of OARs and tumors on patient scans for each RT fraction which requires significant clinician interaction. DL-based approaches are promising solutions to automate this task and reduce burden on clinicians. However, the development of these algorithms is hindered by the paucity of pre-annotated MRI datasets for training and validation. In this study, we developed paired and unpaired training for T_1_W MR image synthesis from pelvic CT scans as a data generative tool for training of segmentation algorithms for MR-Linac RT treatment planning. Our results suggested that the Cycle-GAN network generated synthetic images with the greatest visual fidelity across all pelvic slices whilst the synthetic images from UNet and UNet++ appeared less sharp, which is likely due to soft-tissue misalignments during the registration process. The observed disparity in contrast in Cycle-GAN images for bladder, bone marrow and bowel loops may be due to large variabilities in our relatively small training dataset. Although the direct impact of these contrast discrepancies on MRI segmentation performance is yet to be evaluated, the Cycle-GAN predictions appeared more suitable for CT contour propagation to synthetic MRI than UNet and UNet++ images due to distinctive soft-tissue boundaries and high-resolution synthesis.

Quantitative analysis of all model predictions indicated that the imaging metrics did not fully conform with the output image visual fidelity and apparent sharpness. This finding was in fact in line previous studies comparing paired and unpaired MRI synthesis (12, 22). CT-to-MR synthesis in the pelvis offers the considerable challenge of generating soft-tissue contrasts absent on acquired CT scans. Although quantitative metrics such as the PSNR, SSIM, L_1_ and L_2_ differences are useful measures when comparing images, they may not directly correspond to photo-realistic network outcome. This was evident in quantitative evaluation of the images generated from the UNet and UNet++ models trained with L_1_ loss in the image space against UNet with VGG-19 perceptual loss and Cycle-GAN predictions. Therefore, expert clinician qualitative assessments may provide a more reliable insight into the performance of medical image generative networks. In this study, our expert evaluation test based on Cycle-GAN predictions suggested that despite a number of suboptimal soft-tissue contrast predictions (e.g. urinary bladder filling, bone marrow, nerves), there were differences in radiologist accuracies for correctly identifying synthetic from acquired MR images. The fact that 3/4 radiologists were unable to accurately identify synthetic images in all cases highlights the capability of our model to generate realistic medical images that may be indistinguishable from acquired MRI.

Our segmentation results demonstrated the capability of our fully-automated framework in segmenting bones on acquired MRI images with no manual MR contouring. Domain adaptation offers a significant clinical value in transferring knowledge from previously-contoured OARs by experts on CT to MR-only treatment planning procedures. Additionally, it potentially enables expanding medical datasets which are essential for training supervised DL models. Such a technique is also highly valuable outside the context of radiotherapy, as body MRI has increasing utility for monitoring patients with secondary bone disease from primary prostate (23) and breast (24) cancers, and multiple myeloma (25). Quantitative assessment of response of these diseases to systemic treatment using MRI is hindered by the lack of automated skeletal delineation algorithms to monitor changes in large volume disease regions (26).

GANs are notoriously difficult to train due to their large degree of application-based hyper-parameter optimization and non-standardized training techniques. However, this study showed that even when trained on relatively small datasets, GANs may have the potential to generate realistic images to overcome the challenge of medical image data shortage. Therefore, fut ure studies will investigate the performance of the proposed framework on larger datasets and alternative pelvic OARs, as well as exploring novel techniques to enforce targeted organ contrast during GAN and segmentation training. Additionally, future research will examine the performance sensitivity on the level of manual MRI contours required for training cross-domain DL algorithms.

## Data Availability Statement

The data analyzed in this study is subject to the following licenses/restrictions: The datasets presented in this article are not readily available due to patient confidentiality concerns. Requests to access these datasets should be directed to matthew.Blackledge@icr.ac.uk.

## Ethics Statement

The studies involving human participants were reviewed and approved by the Committee for Clinical Research at the Royal Marsden Hospital. The patients/participants provided written informed consent to participate in this study.

## Author Contributions

All authors listed have made a substantial, direct, and intellectual contribution to the work, and approved it for publication.

## Funding

This project represents independent research funded by the National Institute for Health Research (NIHR) Biomedical Research Centre at The Royal Marsden NHS Foundation Trust and the Institute of Cancer Research, London, United Kingdom.

## Author Disclaimer

The views expressed are those of the authors and not necessarily those of the NIHR or the Department of Health and Social Care.

## Conflict of Interest

The authors declare that the research was conducted in the absence of any commercial or financial relationships that could be construed as a potential conflict of interest.

## Publisher’s Note

All claims expressed in this article are solely those of the authors and do not necessarily represent those of their affiliated organizations, or those of the publisher, the editors and the reviewers. Any product that may be evaluated in this article, or claim that may be made by its manufacturer, is not guaranteed or endorsed by the publisher.
